# Development of a Behavior Change Intervention to Improve Sexual Health Service Use Among University Undergraduate Students: Mixed Methods Study Protocol

**DOI:** 10.2196/resprot.8326

**Published:** 2017-11-02

**Authors:** Christine Cassidy, Audrey Steenbeek, Donald Langille, Ruth Martin-Misener, Janet Curran

**Affiliations:** ^1^ School of Nursing Dalhousie University Halifax, NS Canada; ^2^ Department of Community Health and Epidemiology Dalhousie University Halifax, NS Canada

**Keywords:** sexual health services, university students, sexually transmitted infection, mixed methods research, intervention design, Behaviour Change Wheel, study protocol

## Abstract

**Background:**

University students are at risk for acquiring sexually transmitted infections and suffering other negative health outcomes. Sexual health services offer preventive and treatment interventions that aim to reduce these infections and associated health consequences. However, university students often delay or avoid seeking sexual health services. An in-depth understanding of the factors that influence student use of sexual health services is needed to underpin effective sexual health interventions.

**Objective:**

In this study, we aim to design a behavior change intervention to address university undergraduate students’ use of sexual health services at two universities in Nova Scotia, Canada.

**Methods:**

This mixed methods study consists of three phases that follow a systematic approach to intervention design outlined in the Behaviour Change Wheel. In Phase 1, we examine patterns of sexual health service use among university students in Nova Scotia, Canada, using an existing dataset. In Phase 2, we identify the perceived barriers and enablers to students’ use of sexual health services. This will include focus groups with university undergraduate students, health care providers, and university administrators using a semistructured guide, informed by the Capability, Opportunity, Motivation-Behaviour Model and Theoretical Domains Framework. In Phase 3, we identify behavior change techniques and intervention components to develop a theory-based intervention to improve students’ use of sexual health services.

**Results:**

This study will be completed in March 2018. Results from each phase and the finalized intervention design will be reported in 2018.

**Conclusions:**

Previous intervention research to improve university students’ use of sexual health services lacks a theoretical assessment of barriers. This study will employ a mixed methods research design to examine university students’ use of sexual health service and apply behavior change theory to design a theory- and evidence-based sexual health service intervention. Our approach will provide a comprehensive foundation to co-design a theory-based intervention with service users, health care providers, and administrators to improve sexual health service use among university students and ultimately improve their overall health and well-being.

## Introduction

Progressing from adolescence to adulthood can be a challenging time for young adults who leave home for the first time to start university [1,2]. For most, this transition is uneventful, but for others, newfound independence and campus culture may lead to high-risk behaviors including excessive alcohol consumption [3], casual sex, and inconsistent condom use [4]. It is normal for young adults to explore their sexual identity and sexual relationships throughout their university journey [5]. However, such behaviors can increase students’ risk of undesired health consequences, such as sexually transmitted infections (STIs), unplanned pregnancy, and psychological distress and regret [6]. For example, in Canada, university students are in the age group at highest risk for acquiring an STI [7]. In 2014, the rate of chlamydia infection in young adults in Canada, aged 20-24, was 1627.6 per 100,000 [7].

Many university and college campuses offer a range of sexual health services to promote healthy sexual behaviors (eg, health education, condom distribution) [8] and to prevent sexual health‒related illness (eg, STI/human immunodeficiency virus [HIV] testing and treatment, gynecological exams, pregnancy testing) among students [8,9]. University sexual health services are seen as ideal “health care homes” [8] for students during their studies, as they provide timely, accessible, and convenient services for many students who are away from their primary care provider [8]. However, young adults, including university students, often delay or avoid seeking sexual health care [10-13]. In the United States, only 27% of university students report having ever accessed sexual health services [12].

Based on a review of the literature, Bender and Fulbright [14] identified four categories of perceived barriers to sexual health services among young people in the United Kingdom, United States, and Canada: service access (ie, location, hours, confidentiality), service entry (ie, waiting time, waiting environment, fear of being seen), quality of services (ie, health care provider characteristics), and personal factors (ie, stress associated with seeking sexual health services). Few studies [10,12,13] have examined sexual health service use among the university and college student population specifically, as they begin to explore their sexuality and engage in risky behaviors during their university experience, and found similar results. Enhancing university students’ access to sexual health services is important given the need to prevent their risk of STI transmission and associated negative health consequences [12]. However, we lack a clear understanding of the barriers and enablers to sexual health service use among university students and how their help-seeking behaviors can be changed.

One strategy for addressing students’ use of sexual health services is to use behavior change theory in the design, implementation, and evaluation of sexual health interventions [15].

Incorporation of theory into the development and evaluation of complex interventions facilitates behavior change and provides an explanation of the mechanisms of change [16]. The Medical Research Council [17] in the United Kingdom suggests that complex interventions are more likely to succeed when theory is used to underpin the design process. Many behavioral theories and frameworks exist and have numerous overlapping theoretical constructs, which makes it difficult for researchers to choose a theory and apply it to their behavioral problem. In an effort to make theory more accessible for intervention designers, Michie et al [15,16] developed the Behaviour Change Wheel (BCW). The BCW is a systematic guide to intervention design that is based on (1) an analysis of the target behavior, (2) the determinants of behavior that need to be addressed in order to create behavior change, and (3) the interventions and policies required to support the change [15]. The BCW uses the Capability, Opportunity, Motivation-Behaviour (COM-B) model and Theoretical Domains Framework (TDF) to obtain a better understanding of the behavior in context, which is known as a behavioral analysis. The COM-B model [15] is a theory of behavior that proposes one needs capability (C), opportunity (O), and motivation (M) to perform a behavior. The TDF [18] is a behavioral framework consisting of 14 domains (knowledge, skills, behavioral regulation, beliefs about capabilities, beliefs about consequences, social/professional role and identity, optimism, reinforcement, intentions, goals, memory, attention, and decision making, emotion, environmental context and resources, and social influence) that is used in combination with COM-B to identify specific behavioral determinants of one’s capability, opportunity, and motivation [15]. Based on the behavioral analysis, researchers are guided through a series of systematic steps in the BCW to identify intervention functions, policy categories, and behavior change techniques (BCTs) that are likely to bring about change [15]. The BCW has been used to design interventions in a variety of contexts, such as smoking cessation and alcohol reduction, prescribing behaviors, condom use, and clinician guideline utilization [15].

This paper describes the study protocol for using the BCW to design an intervention to address university undergraduate students’ use of sexual health services at two universities in Nova Scotia, Canada. The study will address the following four research objectives through three phases. Phase 1 will describe the pattern of university undergraduate students’ use of sexual health services at two Nova Scotia universities in 2012 using an existing quantitative dataset. Phase 2 will identify university students’, health care providers’, and university administrators’ perceived barriers and facilitators for student use of sexual health services and will examine how the qualitative data related to the perceived barriers and facilitators to service use help better explain the patterns of student sexual health service use. Phase 3 will identify intervention components and/or strategies that can be used by service providers, university decision makers, policy planners, and students to facilitate the use of sexual health services

## Methods

**Figure 1:**
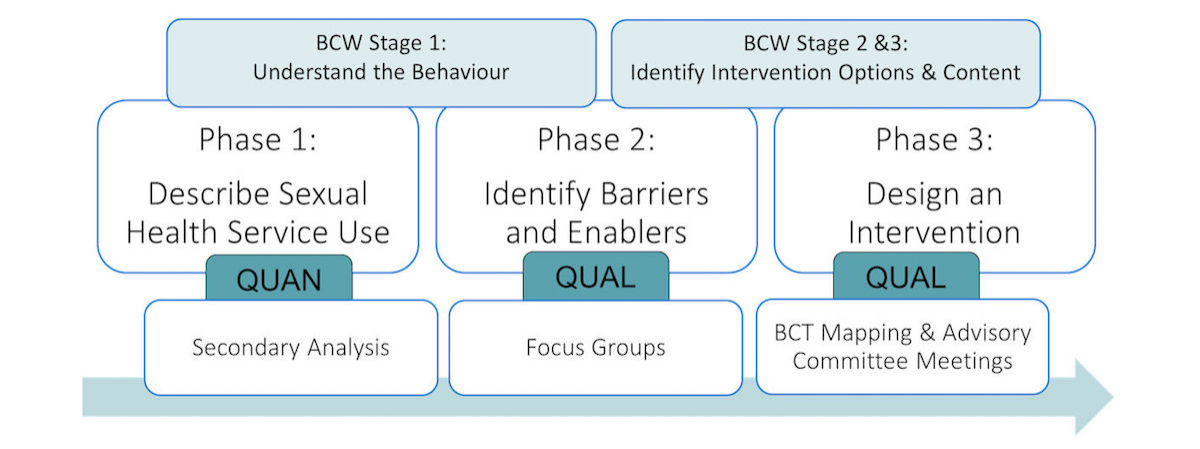
BCW stages and study design diagram.

An explanatory sequential mixed methods research design [19] will be used to address the research objectives (Figure 1). The phases will follow the systematic stages outlined in the BCW. Data gathered from Phases 1 and 2 will be used to guide a series of advisory committee meetings in Phase 3 to identify intervention components that could be used to overcome the barriers and enhance the enablers to sexual health service use. The third phase will culminate in the design of a theory- and evidence-based intervention aimed at improving the use of sexual health services by university students. Future research will pilot test and evaluate this intervention in the university health service setting.

### Phase 1: Understanding the Target Behavior (Quantitative Strand)

#### Design

To understand the pattern of university students’ sexual health service, we will conduct a secondary analysis of data collected during the online Undergraduate Student Sexual Health Survey in the fall of 2012 [20]. This was a cross-sectional survey of a voluntary study population of undergraduate students from eight universities on the east coast of Canada. Data were collected using the Dillman tailored design method [21] through OPINIO, a secure, online surveying service [22]. The survey comprised 49 multiple choice and two open-ended questions. The purpose of this survey was to describe students’ substance use, sexual health knowledge, attitudes, and behaviors, and sexual health service use. We will conduct a secondary analysis of these data to identify significant predictors of students’ sexual health service use.

#### Sample

For the purpose of this study, a secondary analysis of a subset of the data collected from sexually active male and female undergraduate students aged 18-25 at two universities in Nova Scotia will be conducted. Both universities provide general health services in addition to sexual health specific services. These two universities were chosen for three reasons. First, University A is a large urban university, with approximately 13,600 undergraduate students (45% male, 55% female) and University B is a small rural university, with about 3500 undergraduate students (42% male, 58% female) [23]. At University A, 70% of first year undergraduate students and 18% of all undergraduate students live on campus, compared to 77% of first year students and 41% of all undergraduate students at University B [23]. The inclusion of a rural and urban university will improve the generalizability and transferability of the study’s results to universities in similar contexts. Second, as these universities are in relatively close proximity geographically, the data collection in Phases 2 and 3 will be more feasible. Third, University A and University B yielded two of the highest response rates of the eight participating universities (31.2% and 23.8%, respectively; N=5633) [20].

#### Measures

Many factors at the individual, social, service, and policy levels influence young adult and university students’ use of sexual health services [9,12,24,25,26]. The individual- and social-level variables outlined in Table 1 [27-31] were measured in the Undergraduate Student Sexual Health Survey and will be included in the proposed secondary analysis to identify significant predictors of sexual health service use. Survey questions and possible answers can be found in Multimedia Appendix 1.

**Table 1:** Variables of interest for Phase 1 secondary analysis.

Variable of interest	Survey item	Psychometric properties	Composite variable for analyses
Age	What is your age in years?	Pearson correlation =.98 [27]	Continuous variable (18-25)
Ethnic/Racial background	What ethnic/racial background do you consider yourself to be?	New question; no retest performed	0=Caucasian descent (white) 1=non-Caucasian Descent (African Descent, Aboriginal, Asian, Middle Eastern, and other)
Residential status	What are your living arrangements?	New question; no retest performed	0=On campus 1=Off campus, with self or peers 2=Off campus with romantic partner 3=Off campus, with parents
Sexual orientation	People have different feelings about themselves when it comes to questions of being attracted to other people. Which of the following best describes your feelings?	Kappa=.8 [28]	0=Heterosexual 1=Non-heterosexual
Sexual health knowledge [28]	Please indicate whether you believe each of the following statements are true or false by checking the appropriate response.	Cronbach α=.71 [28]	Continuous (0-12)
Barriers to help seeking [29]	Please indicate how much you disagree or agree with the following statements by checking the appropriate number on the 5-point scale, where 1 = “Strongly disagree” and 5 = “Strongly agree”	Cronbach α=.93 [29]	Continuous (0-40)
Social support [30]	Please describe how true you believe each of the following statements about your social relationships and support networks, where 1 = “not true at all” and 5 = “completely true”.	Cronbach α=.86 [30]; .71 [31] Kappa=.91	Continuous variable (0-105)
Sexual health service use Males: STI & HIV testing Females: STI, HIV, Pap, & pregnancy testing	Have you ever seen a health professional in order to obtain the following services? If you answer yes for a particular service, please indicate the location where you access that service: University health center or Other	New question; no retest performed	Males: 0=No 1=Yes (STI or HIV testing) Females: 0=No 1=Yes (STI, HIV, Pap, or pregnancy testing)

#### Data Analysis

Since males and females use sexual health services for different reasons and with different frequencies [6,13,32,33], we will stratify the data by biological sex for all statistical analyses. First, descriptive statistics will be reported to describe the characteristics of the undergraduate students and their use of sexual health services at University A and University B (ie, means/proportions with 95% confidence intervals). Second, to identify factors that are significant predictors of sexual health service use among undergraduate students at the two universities, we will conduct a series of multivariable logistic regressions. We will analyze each of the independent variables using univariable regression to determine significant predictors of sexual health service use at the university health centers. Variables found to be significant predictors (*P*<.2) [32] will be included in multivariable logistic regression analyses using the enter method [34]. For males, a multivariable logistic regression will be conducted with the STI and HIV testing composite dependent variable. For female respondents, a multivariable logistic regression will be conducted with the STI, HIV, Papanicolaou (Pap), and pregnancy testing composite dependent variable (Table 1). We conducted a power analysis and found that a sample size of 5633 is adequate to detect a minimum odds ratio of 1.2 at 89% power. A significance alpha level of *P*<.05 will be used as a cutoff for statistical significance. The data will be analyzed using the statistical software program, SPSS (Statistical Package for the Social Science), Version 21 [35].

#### Anticipated Outputs

Findings from this phase will be used in two ways. First, we will develop a detailed description of the pattern of university undergraduate students’ use of sexual health services on campus. Second, we will incorporate findings into a theory-based semistructured focus group guide to use in Phase 2.

### Phase 2: Understanding the Target Behavior (Qualitative Strand)

#### Design

We will use a qualitative descriptive design [36,37] involving semistructured focus groups and policy document analyses to develop a detailed description of the barriers and facilitators to sexual health service use among university students.

#### Study Population and Sampling

For the focus groups, we will use a stratified purposive sampling strategy [38] with convenience sampling techniques [39] to recruit university undergraduate students, aged 18-25, as well as health care providers and university administrators (ie, health service directors and managers), from the health centers at the two universities. Based on the descriptive results and significant findings from the Phase 1 analysis, we will divide groups of interest into strata (ie, users and nonusers of sexual health services, males and females) and separately recruit participants from each strata to identify their perceived barriers and enablers to sexual health service use. Due to the sensitive nature of the topic, we will conduct single-sex focus groups to facilitate discussion [40]. We will recruit 6-10 participants per focus group as outlined by Wilkinson’s [41] recommendations for conducting focus groups to uncover rich data for health-related phenomena of interest. We aim to recruit 18-30 students from each university (for a total of 36-60) and 6-10 health care providers/university administrators from each university clinic (for a total of 12-20) to participate.

Since the topic of sexual health and use of health services might be a sensitive one for university students [42], recruitment approaches that take place in public places may result in reduced enrollment. As such, we will use recruitment and enrollment mechanisms that allow for discretion. Identical posters and flyers will be distributed across the two university campuses, including libraries and student union buildings. An email describing the study and inviting students to participate will be distributed to student organizations and listservs. For the health care providers and administrator participants, an email will be sent to campus health clinic managers and university administrators with study details and an invitation to participate. Interested participants may contact the research assistant (RA) via email. The RA will respond by sending a study information sheet and a screening questionnaire to student participants to determine eligibility. Once eligibility is confirmed, the RA will send the focus group details and a copy of the consent form. The consent form will be reviewed and completed in person at the focus group meeting. We will provide an option on the consent form for participants to consent to be sent an invitation to participate in the next phase of our research (see Phase 3 below).

#### Materials

We will conduct separate semistructured focus groups with university undergraduate students, health care providers, and university administrators at each university. We will develop a semistructured focus group guide, informed by the COM-B model and TDF to guide the behavioral analysis and probe participants on their perceived barriers and enablers to sexual health service use among university students [43]. This will allow us to identify key beliefs from the different TDF domains that an intervention could target to improve students’ use of sexual health services. As part of the development process, we will review the focus group guide with 3 students and 3 health care providers or administrators. The participants will be asked to read through the guide to identify flaws, uncertainties, concerns about the questions, or need for clarification. The focus groups guides will be refined based on their feedback [44,45].

We chose to conduct semistructured focus groups using a theory-based guide for three reasons. First, focus groups are a useful method for obtaining qualitative data on social and psychological processes [40], as well as social norms and cultural expectations related to sexual health [46]. Second, a semistructured guide will increase the likelihood that participants will cover the topic of interest in an efficient and effective manner [40]. Third, the semistructured guide enables flexibility so the focus group facilitator can explore issues in greater depth [47].

#### Procedure

The principal investigator, who has been trained in conducting focus groups and using the BCW (COM-B and TDF) to conduct behavioral analyses and design interventions, will facilitate the focus groups using the theory-based focus group guide. The focus groups will take place on the university campus and the research assistant will be present to take notes on group dynamics and nonverbal participant observations. Focus groups discussions will be audiorecorded and are expected to last approximately 45-60 minutes. Participants will be offered a Can $30 grocery store gift card in appreciation of their time.

#### Data Analysis

Audiorecordings from the focus groups will be transcribed verbatim and coded using directed content analysis [48] in NVivo 11 [49]. Content analysis is a systematic coding and categorization approach to qualitative data analysis used to examine trends and patterns of the data and to identify the frequency and relationships of the words used by participants [48]. Atkins et al [43] recommend a content analysis approach when using the TDF to design interventions. Focus group transcript analysis will involve the following three steps. First, 2 coders will independently code the first two focus groups by categorizing similar statements into the three COM-B categories and further into the 14 TDF domains. Second, the 2 independent coders will use an inductive coding approach to generate subcategories of specific beliefs of the different groups of participants within the initial coding scheme of the 14 TDF domains. Squires et al [50] define a specific belief as a group of similar responses that suggest the belief may influence the target behavior. The coders will compare their results and examine discrepancies. Discussion will be used to achieve consensus and finalize a coding scheme. All subsequent coding will be guided by the coding scheme in an effort to reduce subjective bias [51]. The 2 coders will independently code all remaining transcripts and meet after every two focus groups to review their coding and seek consensus. Third, the coded data will be further inductively examined to identify relevant theoretical domains to our target behavior [43]. The research team will examine trends, patterns, frequencies, and relationships of the words used by the participants to identify (1) any conflicting beliefs within a domain, (2) the frequency of specific beliefs across the data, and (3) the likely strength of the impact of a belief on the behavior (sexual health service utilization). All three criteria will be considered when examining the relevance of the TDF domains.

#### Policy Document Analysis

Document analysis is a systematic procedure for reviewing documents that involves skimming, reading, and interpreting the text. It is often combined with other qualitative research methods as a way to seek convergence and corroboration or identify inconsistencies and provide data on the context in which the health system operates [31]. We will contact the health clinic managers at University A and University B via email and request a copy of their STI, HIV, Pap, and pregnancy testing guidelines, as well as any general sexual health service policies. Policies will be compared with the current Public Health Agency of Canada screening guidelines [52] to identify differences and similarities between the documents and barriers identified in the focus groups [39].

#### Anticipated Outputs

Findings from this phase will be used in two ways. First, we will use the data to provide a detailed description of students’, health care providers’, and administrators’ perceived barriers and facilitators to sexual health service use among university students. Second, we will use the findings in Phase 3 to develop a theory-based behavior change intervention to address the target behavior (sexual health service utilization).

#### Integration of Quantitative and Qualitative Data

We will integrate the quantitative and qualitative data from Phases 1 and 2 using a triangulation protocol to examine convergence, divergence, and discrepancies from the different data sources [53]. A triangulation protocol is a detailed approach to examine metathemes across findings from different data components that have already been analyzed individually [54]. First, we will create a convergence-coding matrix that will display findings from the quantitative and qualitative phases. Following this, we will evaluate the findings for convergence, divergence, and discrepancies. This approach focuses on explaining the interconnectedness of results between the quantitative and qualitative phases [55]. Overall, by integrating the qualitative and quantitative data, we will generate a clearer understanding of the barriers and enablers to university students’ use and nonuse of sexual health services, which will inform the next phase of intervention design.

### Phase 3: Designing a Theory-based Behavior Change Intervention (Qualitative Strand)

Using the data obtained from Phases 1 and 2, we will develop a theory- and evidence-based intervention that encompasses BCTs aimed at overcoming the identified barriers and enhancing the enablers to sexual health service use by university students. The intervention will be developed through a series of advisory committee meetings which will be guided by Stages 2 and 3 of the BCW. In each meeting, we will use the nominal group technique to generate ideas, identify potential problems, structure the decision-making process, and achieve consensus [56].

#### Step One

The research team will meet to review Phases 1 and 2 findings and identify intervention functions and content. The BCW outlines which types of intervention functions are likely to be effective in bringing about behavior change in each COM-B component and TDF domain [15]. Through discussion, the research team will apply the APEASE criteria (affordability, practicability, effectiveness and cost-effectiveness, acceptability, safety, and equity) to each intervention function to explore its appropriateness for the sexual health service context. The APEASE criteria [15] are used to guide decision making during intervention design. Once the intervention functions are identified, the research team will use the BCT taxonomy (BCTTv1) [57] to identify BCTs that would best serve the intervention function. The BCTTv1 uses a standardized language for describing the active ingredients in interventions [57]. Michie et al [15] developed a matrix that maps relevant BCTs to intervention functions and corresponding COM-B and TDF components. The research team will use the APEASE criteria to consider which BCTs would be feasible within the context of university sexual health service delivery and most useful for addressing the identified barriers and enablers to university students’ use of sexual health services.

#### Step Two

We will form an advisory committee at each university consisting of 3-5 students and 3-5 health care providers and university administrators. Participants who provided consent to be followed up in the Phase Two focus groups will be contacted via email and invited to participate in the advisory committee. The objective of the meeting is to review the findings from Phases 1 and 2 and the results from the BCT mapping exercise (Step One) and further refine the intervention design. Through discussion, the advisory committee will identify potential modes of intervention delivery and apply the APEASE criteria to explore its feasibility. The advisory committee will also discuss optimal intervention content, provider, setting, recipient, intensity, duration, and fidelity.

Following the advisory committee meetings, we will collate the meeting results to produce a summary of the final intervention design that could be delivered in the university setting to improve students’ use of sexual health services. A copy of the intervention design findings will be sent via email to the participants of each advisory committee.

#### Anticipated Outputs

Phase 3 will culminate with a co-designed [58], theory- and evidence-based behavior change intervention for improving sexual health service use among university students.

## Results

Phases 1 and 2 are complete, and Phase 3 intervention design is ongoing. Results from each phase and the finalized intervention design will be reported in 2018.

## Discussion

### Principal Considerations

Increasing university students’ use of sexual health services is important given the need to prevent their risk of STI transmission and associated negative health consequences. This study will follow a systematic, theory-based approach using a mixed methods research design to develop a behavior change intervention aimed at improving university students’ use of sexual health services. The mixed methods approach will allow for an integration of both numerical findings and qualitative text from the perspective of university students, health care providers, and university administrators to enhance our understanding of sexual health service use among university undergraduate students. This study is guided by the BCW, which uses the COM-B model and TDF as theoretical approaches to understanding the target behavior in context and designing theory-based interventions. The BCW has been used extensively in health services research [59-61], including the design of sexual health‒related interventions for young adults [62,63]. Based on the success of these studies, we anticipate the proposed theory- and evidence-based intervention will be successful at improving university undergraduate students’ use of sexual health services.

### Limitations

All findings from this study will be interpreted with the following limitations in mind, among others that may arise. First, the two universities included in the Phase 1 secondary analysis had response rates of 31.2% and 23.8%. These response rates are lower than the primary researchers had anticipated, as previous Web-based survey research with Canadian university students had a mean response rate of 40.9%. Further, Web-based sexual health research with US college students yielded response rates that ranged from 24% to 55%. This can result in nonresponse bias that may impact generalizability of the study findings. Second, the Phase 1 data were collected in 2012, which may result in findings that are no longer relevant today. For example, with recent developments in health service technologies (eg, online booking, electronic notification of results, online provision of sexual health information), there may be differences in the accessibility and acceptability of sexual health services among university students. However, our Phase 2 focus groups with students, health care providers, and university administrators will provide an opportunity to follow up on the 2012 data and describe any differences in the accessibility and acceptability of sexual health services during this period of time. Last, a limitation of secondary analyses is that researchers must work with the available data, which may not have been collected to address the research question. The only measures of sexual health service use in the secondary dataset are STI testing, HIV testing, Pap testing, and pregnancy testing. The Phase 2 focus groups will allow for further exploration of a more comprehensive definition of sexual health services, including sexual health promotion initiatives.

### Conclusion

Overall, the methods presented in this paper demonstrate a theoretically robust and evidence-based approach to design an intervention to improve university students’ use of sexual health services. The BCW will be used to understand the behavior in greater detail, identify intervention options, content, and implementation strategies. Future pilot testing in university settings will be needed to evaluate the effectiveness of the proposed intervention.

## Appendix

Multimedia Appendix 1 Survey questions.

Multimedia Appendix 2 Existing Peer-Review Report.

## Abbreviations

BCT: behavior change techniques
BCTTv1: Behaviour Change Technique Taxonomy Version 1
BCW: Behaviour Change Wheel
TDF: Theoretical Domains Framework
