# Supplementary material for: Development of a Behavior Change Intervention to Improve Sexual Health Service Use Among University Undergraduate Students: Mixed Methods Study Protocol
Source: JMIR Res Protoc. 2017 Nov 2;6(11):e217. doi: 10.2196/resprot.8326 (PMC5691239; doi:10.2196/resprot.8326)
Supplement: Multimedia Appendix 1 [file resprot_v6i11e217_app1.pdf]

Undergraduate Health Survey Questions

Demographic Variables:

2. What is your age in years? ___________________

3. What ethnic/racial background do you consider yourself to be? (*Check all that apply.)*
   - ☐ White (Caucasian)
   - ☐ African descent
   - ☐ Aboriginal (*specify*) _______________________________________
   - ☐ Asian
   - ☐ Middle Eastern
   - ☐ Other (*describe*) _______________________________________

7. Who do you live with?
   - ☐ I live alone
   - ☐ I live with one or both of my parent(s)
   - ☐ I live with my partner (i.e., sexual or romantic partner, spouse or girlfriend/boyfriend)
   - ☐ I live with a roommate(s) (not a sexual or romantic partner)

10. What is your sex?

☐ Male ☐ Female ☐ Transgendered ☐ Other (*describe*) _________________________________

11. People have different feelings about themselves when it comes to questions of being attracted to other people. Which of the following best describes your feelings?

☐ 100% heterosexual (attracted to persons of the opposite sex)

☐ Mostly heterosexual

☐ Bisexual (attracted to both males and females)

☐ Mostly homosexual

☐ 100% homosexual (gay/lesbian, attracted to persons of the same sex)

☐ Transgendered

☐ Not sure

Sexual Health Knowledge

14. Please indicate whether you believe each of the following statements are true or false by checking the appropriate response. If you do not know the answer, please do not guess, but answer "Don't Know".

	True	False	Don't Know
If you know a person's sexual history and lifestyle before you have sex with them, you don't need to use condoms	☐	☐	☐
Men with chlamydia always have symptoms	☐	☐	☐
Women with chlamydia always have symptoms	☐	☐	☐
Chlamydia infection in women can result in being unable to have children	☐	☐	☐
If a guy or girl aged 18 – 24 gets chlamydia and is treated properly, he or she can never get chlamydia again	☐	☐	☐
If both are used properly, condoms are just as effective as birth control pills in preventing pregnancy	☐	☐	☐
Emergency contraceptive pills are available at pharmacies	☐	☐	☐
Emergency contraceptive pills always prevent pregnancies	☐	☐	☐
To be effective, emergency contraceptive pills must be taken within 12 hours of unprotected sex	☐	☐	☐
Emergency contraceptive pills are more effective the earlier they are taken after unprotected sex	☐	☐	☐
Doctors will always test for STIs when they do a PAP test	☐	☐	☐
The time in the monthly menstrual cycle during which a female is most likely to become pregnant is about two weeks before her period begins	☐	☐	☐

Social Support

18. Please describe how true you believe each of the following statements about your social relationships and support networks, where 1 = not at all true and 5 = completely true

	1	2	3	4	5
**I participate in volunteer/service projects**	☐	☐	☐	☐	☐
**I have meaningful conversations with my parents and or/siblings**	☐	☐	☐	☐	☐
**I have a mentor(s) in my life I can go to for support/advice**	☐	☐	☐	☐	☐
**I seldom invite others to join me in my social and or/recreational activities**	☐	☐	☐	☐	☐
**There is at least one person I feel a strong emotional tie with**	☐	☐	☐	☐	☐
**There is no one I can trust to help solve my problems**	☐	☐	☐	☐	☐
**I take time to visit my neighbours**	☐	☐	☐	☐	☐
**If a crisis arose in my life, I would have the support I need from family and/or friends**	☐	☐	☐	☐	☐
**I belong to a club (e.g., sports, hobbies, support group, special interests)**	☐	☐	☐	☐	☐
**I have friends from work that I see socially (movie, dinner, sports etc)**	☐	☐	☐	☐	☐
**I have friendships that are mutually fulfilling**	☐	☐	☐	☐	☐
**There is no one I can talk to when making important decisions in my life**	☐	☐	☐	☐	☐
**I make an effort to keep in touch with friends**	☐	☐	☐	☐	☐
**My friends and family feel comfortable asking me for help**	☐	☐	☐	☐	☐
**I find it difficult to make new friends**	☐	☐	☐	☐	☐
**I look for opportunities to help and support others**	☐	☐	☐	☐	☐
**I have a close friends(s) who I feel comfortable sharing deeply about myself**	☐	☐	☐	☐	☐
**I seldom get invited to do things with others**	☐	☐	☐	☐	☐

I feel well supported by my friends and/or family	☐	☐	☐	☐	☐
I wish I had more people in my life that enjoy the same interests and activities as I do	☐	☐	☐	☐	☐
There is no one that shares my beliefs and attitudes	☐	☐	☐	☐	☐

Barriers to Help-Seeking

19. Please indicate how much you disagree or agree with the following statements by checking the appropriate number on the 5 point scale, where 1 = “Strongly disagree” and 5 = “Strongly agree”.

	1	2	3	4	5
**I would think less of myself for needing help**	☐	☐	☐	☐	☐
**I don’t like other people telling me what to do**	☐	☐	☐	☐	☐
**Nobody knows more about my problems than I do**	☐	☐	☐	☐	☐
**I’d feel better about myself knowing I didn’t need help from others**	☐	☐	☐	☐	☐
**I don’t like feeling controlled by other people**	☐	☐	☐	☐	☐
**It would seem weak to ask for help**	☐	☐	☐	☐	☐
**I like to make my own decision and not be too influenced by others**	☐	☐	☐	☐	☐
**Asking for help is like surrendering authority over my life**	☐	☐	☐	☐	☐

Sexual Health Service Use

47. Have you ever seen a health professional in order to obtain the following services? If you answer ‘Yes’ for a particular service, please indicate the location where you access that service.

**Service:**	**Accessed?**		**If yes, please indicate location**	
	**Yes**	**No**	**University health centre**	**Other**
STI testing	☐	☐	☐	☐
PAP testing	☐	☐	☐	☐
HIV/AIDS testing	☐	☐	☐	☐
Pregnancy testing	☐	☐	☐	☐
