# Supplementary material for: Development of a Behavior Change Intervention to Improve Sexual Health Service Use Among University Undergraduate Students: Mixed Methods Study Protocol
Source: JMIR Res Protoc. 2017 Nov 2;6(11):e217. doi: 10.2196/resprot.8326 (PMC5691239; doi:10.2196/resprot.8326)
Supplement: Multimedia Appendix 2 [file resprot_v6i11e217_app2.pdf]

Scientific Officer Report

May 2016 Competition, Nursing Research & Development Fund

Title: **Using a mixed methods approach to design an intervention to improve sexual healthcare service use among university undergraduate students in Nova Scotia**

Pi: Christine Cassidy

Scoring and Comments

3 reviewers reviewed the application. The score was 48.5 out of a maximum of 50 which was in the fundable range.

Below are the reviewers' comments that we hope you will find helpful but we want to clarify that they are not a requirement nor condition for funding.

Reviewer 1: Not going to be submitted for funding now but informs PhD Phase 3 – then ongoing research program

Reviewer 2: Very strong literature review that provides great background and context for the study and its importance. Applicant needs to indicate clearly why students from Acadia was selected, along with Dalhousie students, as participants, as opposed to Saint Mary's or the Mount.

It is not clear in the proposal or budget justification the exact number of participants that will participate in this study since the applicant states "up to 46 students" in various sections of the proposal. Applicant needs to provide the exact number of participants in the study and how many students, administrators and clinicians will participate in each focus group. While the Budget section indicates that there will 10 focus groups, the Summary of Proposal and the main proposal do not indicate clearly the number of focus groups or how many individuals from each participant category (students, admin, clinicians) will participate in each focus group. Proposal also does not indicate how many participants from Acadia and Dalhousie will be recruited. Applicant may also want to provide information on any unique or distinguishing factors between Acadia students and Dalhousie students.

Applicant does not clearly indicate impediments to completion of the study.

Applicant does not provide sufficient details about the Behaviour Change Theory in the Background and Literature Review section. While the applicant does a great job of discussing the benefits of this Theory, she does not provide sufficient information on the theory itself.

External: The proposal is generally clear. The project itself lacks some methodological rigor in terms of theory. The behavioural change model, while somewhat appropriate for understanding individual health issues, holds less potential to impact either population health or a significant number of university students. The number of focus groups suggested seems to be high and difficult to accomplish. It is unlikely that service providers will be available at the same time to conduct a focus group.

The PI has outlined a relatively good plan for completing the project, but it may not be realistic. Are service providers willing/able to commit to a focus group etc. Further, while the objectives and purpose of the study are evident, the various challenges that may be encountered are not completely considered. How will students respond in focus groups to questions about their sexual practices in a fashion that address the barriers and facilitators to access?

The researcher and supervisors have a strong record of publication and collaboration. The PI and team are well-suited to carry out the proposed research.

Overall, the proposed research is well-thought out and clearly articulated.

Comments on Budget

Reviewer 1: All eligible – mostly honoraria

Reviewer 2: Budget seems reasonable, however, it is not clear in the proposal or budget justification exactly how many participants will participate since the applicant states "up to 46 students...". Applicant needs to indicate exact number of participants and calculate budget based on that number

External: No comments
